# 肺癌化疗合并院内肺部感染的病原菌特点及影响因素

**DOI:** 10.3779/j.issn.1009-3419.2019.12.07

**Published:** 2019-12-20

**Authors:** 秋红 鲍, 华 周, 茜 陈, 青 杨, 建英 周

**Affiliations:** 1 310003 杭州，浙江大学医学院附属第一医院呼吸与危重症医学科 Department of Respiratory Disease, Thoracic Disease Center, The First Affiliated Hospital, College of Medicine, Zhejiang University, Hangzhou 310003, China; 2 317200 天台；浙江省天台县人民医院呼吸与危重症医学科 Department of Respiratory and Critical Medicine, Zhejiang Tiantai People's Hospital, Tiantai 317200, China

**Keywords:** 肺肿瘤, 化疗, 肺部感染, 病原菌, 影响因素, Lung neoplasms, Chemotherapy, Pulmonary infection, Pathogenic bacteria, Influence factor

## Abstract

**背景与目的:**

本研究旨在探讨肺癌患者化疗期间并发院内肺部感染的病原菌类型、分布、耐药性及影响因素。

**方法:**

本研究回顾性分析2017年1月-2018年12月于浙江大学医学院附属第一医院住院治疗的411例肺癌化疗患者的临床资料，统计患者院内肺部感染发生率、病原体、耐药性及影响因素。

**结果:**

411例肺癌患者在化疗期间发生院内肺部感染184例次，感染率达44.77%，分离的病原体包括革兰阴性菌、革兰阳性菌、病毒、真菌和结核，其中以革兰阴性菌为主，占37.25%，其次为病毒感染，占15.69%。革兰阴性菌以铜绿假单胞菌和肺炎克雷伯菌为主，革兰阳性菌以金黄色葡萄球菌和肺炎链球菌常见，病毒以乙型流感病毒为主，真菌以念珠菌和曲霉菌多见。铜绿假单胞菌对亚胺培南的耐药率偏高，达26.67%，而肺炎克雷伯菌对亚胺培南的耐药率仍保持低水平，为12.50%；主要革兰阳性菌对万古霉素的耐药率均为0.00%。低蛋白血症、化疗周期长、高强度化疗及合并有慢性阻塞性肺疾病、支气管扩张基础疾病者是肺癌患者在化疗期间合并院内肺部感染的高危因素（*P* < 0.05）。

**结论:**

肺癌患者化疗期间合并院内肺部感染的病原菌分布和耐药性具有一定的特征，临床医生应加强对病原菌及其耐药性检测，在对症治疗的基础上，以达到保障治疗效果、延长患者生存期的目的，对高危患者应做好预防措施，尽可能降低化疗周期及强度，以减少感染的发生。

## Abstract (en)

**Background and objective:**

The aim of this study is to explore the pathogenic bacteria type, distribution, drug resistance and influencing factors of nosocomial pulmonary infection in patients with lung cancer during chemotherapy.

**Methods:**

This study retrospectively analyzed the clinical data of 411 patients with lung cancer who were hospitalized in the First Affiliated Hospital of Zhejiang University Medical College from January 2017 to December 2018, and counted the incidence of nosocomial lung infection, pathogens, drug resistance and influencing factors.

**Results:**

There were 184 cases of nosocomial pulmonary infection in 411 lung cancer patients during chemotherapy, the infection rate was 44.77%. The isolated pathogens included Gram-negative bacteria, Gram-positive bacteria, viruses, fungi and tuberculosis, among which Gram-negative bacteria accounted for 37.25%, followed by virus infection, accounting for 15.69%. *Pseudomonas aeruginosa* and *Klebsiella pneumoniae* are the main Gram-negative bacteria, *Staphylococcus aureus* and *Streptococcus pneumoniae* are the common gram-positive bacteria, influenza B virus is the main virus, Candida and Aspergillus are the most common fungi. The resistance rate of *Pseudomonas aeruginosa* to imipenem was 26.67%, while that of *Klebsiella pneumoniae* to imipenem was 12.50%, and that of the main Gram-positive bacteria to vancomycin was 0.00%. Hypoproteinemia, long chemotherapy cycle, high-intensity chemotherapy, chronic obstructive pulmonary disease and basic bronchiectasis were the high risk factors of lung cancer patients with nosocomial pulmonary infection during chemotherapy (*P* < 0.05).

**Conclusion:**

During the chemotherapy of lung cancer patients with nosocomial pulmonary infection, the distribution and drug resistance of pathogenic bacteria have certain characteristics. Clinicians should strengthen the detection of pathogenic bacteria and their drug resistance. On the basis of symptomatic treatment, to achieve the purpose of ensuring the treatment effect and prolonging the survival period of patients, preventive measures should be taken for high-risk patients to reduce the chemotherapy cycle and intensity as much as possible to reduce the incidence of infection life.

肺癌又称原发性支气管肺癌，指的是起源于支气管黏膜上皮的恶性肿瘤。恶性肿瘤（癌症）已经成为严重威胁中国人群健康的主要公共卫生问题之一，2019年1月，国家癌症中心发布了最新一期的全国癌症统计数据。肺癌位居男性发病第1位，每年新发病例约52.0万，按发病人数顺位排序，肺癌位居我国恶性肿瘤发病首位。估计结果显示，2015年我国新发肺癌病例约为78.7万例，发病率为57.26/10万，中标率为35.96/10万。按死亡人数顺位排序肺癌位居我国恶性肿瘤死亡第1位，2015年我国因肺癌死亡人数约为63.1万例，死亡率为45.87/10万，中标率为28.16/10万^[1]^。2019年美国癌症统计：新发癌症中肺癌仍排第2位，且肺癌死亡病例将占所有癌症死亡的1/4^[2]^。而肺癌起病隐匿，容易被患者忽略而失去早期手术治疗的机会，一旦确诊多为中晚期，治疗效果和预后差^[3]^。尽管随着医疗技术发展，分子靶向药物、免疫治疗等多元化、个体化治疗方案成为中晚期肺癌的主要手段，但目前化疗仍为基石，术后、基因野生型、程序性死亡受体1（programmed cell death-1, PD-1）和程序性死亡受体1配体（programmed cell death-1 ligand, PD-L1）低表达者，化疗仍为一线治疗方案。但化疗不仅带来了不良反应和各种损害患者身心的并发症如肝肾损害和胃肠道症状等，还使得患者的免疫力出现不同程度的下降而合并感染^[4]^。感染不但影响了患者的生存质量，还加重了患者的经济负担，患者预后一般较差。为进一步探讨肺癌化疗患者合并院内肺部感染的相关因素，分析其病原菌分布特点及耐药性，为临床医师合理用药提供参考，本研究对近年收治的411例肺癌化疗患者的临床资料进行分析，现将研究结果报道如下。

## 1 资料与方法

### 1.1 研究对象

选取浙江大学附属第一医院2017年1月-2018年12月收治的411例Ⅰ期-Ⅳ期肺癌化疗的临床资料进行回顾性分析，其中184例院内肺部感染（其中腺癌64例，鳞癌87例，小细胞癌34例），男性159例、女性25例；年龄21岁-87岁，平均（55.29±6.12）岁，合并慢性阻塞性肺疾病56例，合并支气管扩张11例，合并低蛋白血症30例。

### 1.2 诊断及纳入标准

#### 1.2.1 纳入标准

均为本院从2017年1月-2018年12月收治的肺癌患者，治疗过程中均接受化疗治疗；有肺癌的组织病理学依据，根据《第八版国际肺癌原发灶-淋巴结-远处转移（tumor-node-metastasis, TNM）分期》^[5]^诊断；患者肺部感染诊断标准参照国家卫生计生委颁布的《医院感染诊断标准》〔试行〕^[6]^。

#### 1.2.2 诊断标准

临床诊断：（1）患者出现咳嗽、痰黏稠，肺部出现湿啰音，并有以下情况之一：①发热；②白细胞总数和（或）嗜中性粒细胞比例增高；③X线显示肺部有炎性浸润性病变。（2）慢性气道疾病患者稳定期（慢性支气管炎伴或不伴阻塞性肺气肿、哮喘、支气管扩张症）继发急性感染，并有病原学改变或X线胸片显示与入院时比较有明显改变或新病变；病原学诊断：（1）经筛选的痰液，连续2次分离到相同病原体。（2）痰细菌定量培养分离病原菌数≥10^6^ cfu/mL。经纤维支气管镜或人工气道吸引采集的下呼吸道分泌物病原菌数≥10^6^ cfu/mL；经支气管肺泡灌洗（bronchoalveolar lavage, BAL）分离到病原菌数≥10^4^ cfu/mL；或经防污染标本刷（protected specimen brush, PSB）、防污染支气管肺泡灌洗（protected bronchoalveolar lavage, PBAL）采集的下呼吸道分泌物分离到病原菌，而原有慢性阻塞性肺疾病合并支气管扩张者病原菌数必须≥10^3^ cfu/mL。

### 1.3 试验方法

（1）采集痰液：采集全部患者清晨盐水漱口后深咳痰液及支气管镜肺泡灌洗液两种样本于无菌容器中并及时送检，进行细菌培养和药敏试验以及病毒核酸反转录-聚合酶链反应（reverse transcription-polymerase chain reaction, RT-PCR）检测，全自动细菌鉴定及药敏分析系统使用生物梅里埃公司的全自动微生物生华鉴定仪VITEK-2 Compact，采用K-B纸片法进行药物敏感试验，结果参照美国临床实验室标准化委员会（National Committee for Clinical Laboratory Standards, NCCLS）标准^[7]^。

### 1.4 统计学方法

数据均由Excel软件以及SPSS 19.0软件进行处理，统计差异运用t或卡方检验，当*P* < 0.05为差异有统计学意义；药敏数据使用WHONET 5.3软件进行统计。

## 2 结果

### 2.1 医院感染率及易感因素

411例肺癌患者中发生院内感染184例，感染率44.77%，男性159例，女性25例；184例院内感染，患者影响因素见表 1。

**1:** 肺癌化疗中发生院内肺部感染因素分析 Analysis of factors causing nosocomial pulmonary infection in the lung cancer chemotherapy

Category	Total	Infected patients	No infected patients	*χ*^2^	*P*
Age (yr)				0.376	0.542
≥65	212	98	114		
<65	199	86	113		
Gender				0.001	0.981
Male	364	159	205		
Female	57	25	32		
Stage				1.525	0.217
Ⅲ-Ⅳ	349	156	193		
Ⅰ-Ⅱ	52	28	24		
Diabetes				3.651	0.056
Yes	18	12	6		
No	393	172	221		
COPD				13.293	0.000
Yes	91	56	35		
No	320	128	192		
Anemia				0.133	0.716
Yes	45	19	26		
No	366	165	201		
Bronchiectasia				3.872	0.049
Yes	16	11	5		
No	395	173	222		
Hypoproteinemia				7.798	0.005
Yes	47	30	17		
No	364	154	210		
Chemotherapeutics (typ)				8.488	0.003
≤2	228	71	157		
≥3	183	82	101		
Chemotherapy (cycle)				5.921	0.015
≤2	126	43	83		
≥3	285	134	151		
Pathological type				0.095	0.757
NSCLC	315	151	164		
SCLC	68	34	34		
NSCLC: non-small cell lung cancer; SCLC: small cell lung cancer; COPD: chronic obstructive pulmonary disease.					

### 2.2 病原菌分布及构成比

184例肺炎患者采集的符合合格痰液/支气管肺泡灌洗液进行培养，共检出204株病原菌，见表 2。

**2:** 患者感染病原菌分布构成比 Composition ratio for pathogen distribution for all patients infected

Pathogenic bacteria	*n*	%
Gram negative bacteria	84	41.18
*Klebsiella pneumonia*	16	7.84
*Acinetobacter baumannii*	10	4.90
*Pseudomonas aeruginosa*	30	14.71
*Escherichia coli*	2	0.98
*Oligotrophic maltophilia*	8	3.92
*Haemophilus influenza*	8	3.92
*Acinetobacter Pitti*	2	0.98
*Serratia rubidaea*	4	1.96
*Bacillus subtilis*	2	0.98
*Ralstonia caries*	2	0.98
Gram positive bacteria	32	15.68
*Staphylococcus aureus*	18	8.82
*Streptococcus pneumonia*	14	6.86
Fungus	36	17.65
Candida glabrata	12	5.88
Candida tropicalis	10	4.90
Aspergillus	10	4.90
Cryptococcus	4	1.96
Viruses	44	21.57
Influenza virus	32	15.69
Cytomegalovirus	4	1.96
Respiratory syncytial virus	8	3.92
Mycobacterium tuberculosis	8	3.92
Total	204	100.00

### 2.3 耐药率分析

病原菌对抗菌药物耐药性结果见表 3、表 4。

**3:** 主要革兰阴性菌对抗菌药物的耐药率 Drug resistance rate of mainly Gram-negative bacteria to antimicrobials

Antibacterial drugs	Drug resistance rate (%)			
	*Klebsiella pneumoniae* (*n*=16)	*Acinetobacter baumannii* (*n*=10)	*Pseudomonas aeruginosa* (*n*=30)	*Pseudomonas maltophilia* (*n*=8)
Amikacin	12.50	20.00	0.00	-
Aztreonam	-	40.00	46.67	-
Ciprofloxacin	25.00	40.00	20.00	-
Cefatriaxone	25.00	40.00	-	-
Gentamicin	-	40.00	-	-
Imipenem	12.50	40.00	26.67	-
Levofloxacin	37.50	40.00	20.00	25.00
SMZ	0.00	20.00	-	0.00
Tobramycin	-	20.00	20.00	-
Cefepime	-	40.00	20.00	-
Piperacillin sodium and Tazobactam sodium	25.00	40.00	20.00	-
Tegafycline	0.00	0.00	-	-
Ertapenem	12.50	40.00	-	-
Cefazolin	62.50	40.00	-	-
Cefoxitin	50.00	40.00	-	-
Amoxicillin clavulanic acid	37.50	40.00	-	-
Furofen	12.50	40.00	-	-
Meropenem	12.50	40.00	20.00	-
Ceftazidime	12.50		26.67	-
Cefoperazone and sulbactam	25.00	40.00	20.00	0.00
Latamoxef	12.50	40.00	-	-
Ampicillin	50.00	40.00	-	-
Minocycline	6.25	40.00	-	0.00
Colistin	-	0.00	0.00	-

**4:** 主要革兰阳性菌对抗菌药物的耐药率 Drug resistance rate of mainly gram-positive to antimicrobials

Antibacterial drugs	Drug resistance rate (%)	
	*Streptococcus pneumoniae* (*n*=14)	*Staphylococcus aureus* (*n*=18)
Rifampin	0.00	0.00
Tegafyclin	0.00	0.00
Linezolid	-	0.00
Furofen	0.00	0.00
Gentamicin	0.00	28.57
Quinupristin/Dalfopristin	33.33	28.57
Tetracycline	0.00	14.28
Vancomycin	0.00	0.00
Moxifloxacin	0.00	57.14
Clindamycin	100.00	42.86
Ciprofloxacin	0.00	42.86
Erythromycin	100.00	42.86
Levofloxacin	33.33	71.43
Oxacillin	0.00	71.43
Penicillin G	0.00	85.71
Teicoplanin	0.00	0.00

## 3 讨论

晚期非小细胞肺癌（non-small cell lung cancer, NSCLC）的一线治疗多应用靶向治疗或含铂双药化疗或化疗联合免疫等治疗，靶向药物耐药后仍需二线化疗治疗。目前在肺癌发生及发展过程中，包括肺部感染等并发症纷纷出现，对肺癌患者的临床治疗造成了极大影响，甚至成为直接造成患者死亡的原因^[8]^。化疗的主要作用机制是促使肿瘤细胞分化、抑制肿瘤细胞的生长繁殖以及杀死恶性肿瘤细胞，属于一种全身性治疗手段，其对原发性病灶、亚临床转移病灶及转移性病灶均有一定疗效^[9]^。既往有研究^[10]^报道，老年肺癌患者在接受化疗的过程中，由于老年患者免疫力下降、肿瘤局部出现阻塞及化疗对骨髓的抑制作用等方面的原因，容易并发感染。下呼吸道是医院感染中最常见的感染部位，主要由细菌、真菌、病毒、支原体引起，在发展中国家以细菌感染为主^[11]^。肺癌患者化疗后，因自身基础条件差，免疫力低下更明显，因此易出现下呼吸道感染。本次调查研究显示：低蛋白血症、合并有慢性阻塞性肺疾病、支气管扩张基础疾病者、经过3种以上药物化疗或化疗周期超过3个周期以上者是肺癌患者在化疗期间合并院内肺部感染的高危因素（*P* < 0.05），而性别、年龄、分期、分型与关系不大，与相关研究^[12]^结果基本相符。

本次研究中显示184例肺癌合并院内肺部感染患者的临床资料，以革兰阴性菌感染为主，共84株，占41.18%，其中以铜绿假单胞菌最为多见，肺炎克雷伯菌次之；病毒感染共44株，占21.57%，以流感病毒为主，共32株，占15.69%；全球及全国流感监测结果^[13]^显示，2017年入冬以来流感患者就诊比例及流感病毒检测阳性率均高于过去3年同期水平，流感活动强度高于往年，主要病原体是甲型和乙型流感，本研究与此结果相符合。革兰阳性菌32株，占15.68%，真菌36株，占17.65%，结核杆菌8例，占3.92%。

本研究对病原菌耐药分析，结果显示革兰阴性菌中：铜绿假单胞菌耐药率较高，对亚胺培南耐药率达26.67%，且发生铜绿假单胞菌感染患者均有慢性阻塞性肺疾病或支气管扩张症基础疾病，多重耐药考虑与基础慢性肺组织结构性破坏、反复感染应用抗菌药物治疗、反复住院等因素有关，符合相关文献^[14]^报道。肺炎克雷伯氏杆菌对头部唑林/氨苄西林基本耐药，对左氧氟沙星/阿莫西林克拉维酸/头孢他定/头孢西丁大部分耐药，对环丙沙星/头孢哌酮舒巴坦/哌拉西林他唑巴坦部分耐药，对美罗培南/厄他培南/亚胺培南/阿米卡星仍保持较低耐药率，仅为12.5%，令人欣慰的是对替加环素/复方新诺明均敏感。该研究还显示鲍曼杆菌多重耐药多见，对亚胺培南、头孢哌酮/舒巴坦等耐药率达40%，这与鲍曼不动杆菌具有强大的获得耐药性和克隆传播能力有关，据报道^[15]^多重耐药、广泛耐药、全耐药鲍曼不动杆菌呈世界性流行，已成为我国院内感染最重要的病原菌之一。本研究显示在革兰阳性球菌中：金黄色葡萄球菌对青霉素G高度耐药，耐药率达85.71%，但所幸对替加环素、利柰唑胺、万古霉素未产生耐药。而肺炎链球菌对青霉素G敏感性仍保持较高。真菌中白色假丝酵母菌对5-氟尿嘧啶、两性霉素B的耐药率为0.00%，曲霉菌对伊曲康唑/伏立康唑高度敏感，隐球菌对氟康唑均敏感。

根据相关文献研究结果^[16]^显示，350例肺癌患者发生术后肺部感染34例，感染率为9.71%，主要病原菌为革兰阴性菌，共有42株，其中最常见的是肺炎克雷伯菌，占57.81%。对106例肺癌姑息治疗合并肺部感染的患者痰液标本进行病原菌的培养，共培养出病原菌174株，发现主要的感染病原菌亦为革兰阴性菌，占58.62%，检出革兰阴性菌以铜绿假单胞菌和肺炎克雷伯菌等为主，分别占21.26%和17.24%^[17]^。对比研究结果，肺癌化疗及姑息治疗并发肺部感染的感染率明显偏高，但三者病原菌分布基本雷同，主要的感染病原菌为革兰阴性菌，均为条件致病菌多见，与肺癌患者手术、化疗及病情进展为晚期时机体抵抗力下降容易诱发有关。

综上，肺癌化疗治疗后合并院内肺部感染的感染率较高，在患者的病原菌的主要类型为革兰阴性菌，以条件致病菌为主，并在流感季节，易被感染，真菌和结核感染不能被忽视。不论是革兰阴性杆菌还是革兰阳性球菌都表现出很高的耐药性，故临床应尽早进行相关病原菌的检查，如痰、肺泡灌洗液或血等培养及药敏试验，根据不同的病原菌的耐药性，选择合适的敏感抗菌药物，合理联合用药。另外需要重视患者原发病的治疗，需要建立配套的消毒灭菌措施，培养无菌操作意识，最大程度地减少耐药菌的出现。在流感病毒流行季节，可提前注射流感疫苗，减少流感发生。最后我们最好对当地医院呼吸道感染的病原学进行监测，了解地区病原菌分布及耐药情况，为减少细菌耐药性提供科学依据。肺癌患者需密切关注低蛋白血症情况，适当加强营养，增强免疫力，减少条件致病菌发生机会。在选择治疗方案时尽可能选用低强度药物化疗方案，在长周期化疗中密切观察体温、咳嗽咳痰、炎症指标等肺部感染症状，以便及时抗感染治疗，改善患者生存质量。

## Author contributions

Zhou JY conceived and designed the study. Bao QH performed the experiments. Zhou H, Yang Q and Bao QH analyzed the data. Chen X contributed analysis tools. Zhou JY, Zhou H, Bao QH and Yang Q provided critical inputs on design, analysis, and interpretation of the study. All the authors had access to the data. All authors read and approved the final manuscript as submitted.
